# Optimizing joint engagement in autism: considering toddler developmental abilities and caregiver stress

**DOI:** 10.3389/frcha.2026.1858019

**Published:** 2026-07-20

**Authors:** Sydney Seese, Angelina Matar, Lily Mendes, Amanda Gulsrud, Connie Kasari

**Affiliations:** Semel Institute for Neuroscience and Human Behavior, University of California Los Angeles, Los Angeles, CA, United States

**Keywords:** autism spectrum disorder, caregiver-mediated early intervention, joint engagement, language development, toddler

## Abstract

**Introduction:**

Play interactions between caregivers and their toddlers provide a critical context for the emergence of joint engagement, a state that supports early learning andsocial communication. Differences in toddler's developmental characteristics and caregiver experiences may influence how joint engagement is established during these interactions. This study examined whether toddler characteristics, including age, cognitive abilities, language abilities (expressive and receptive), and caregiver stress attenuated or amplified the association between caregiver strategy use and joint engagement in toddlers with autism.

**Methods:**

Participants include 80 toddlers with autism (*M* age = 26.26 months) and their caregivers. Toddler cognitive and language abilities were assessed at baseline (prior to any intervention) using the Mullen Scales of Early Learning, and caregiver stress was measured using the Parenting Stress Index. Caregiver strategies and child engagement were observationally coded during a 10-min caregiver-child free-play interaction.

**Results:**

Results indicated that age, cognitive abilities, expressive and receptive language significantly moderated the association between caregiver strategy use and joint engagement. The positive association between higher quality caregiver strategies and joint engagement was stronger for toddlers who were older and had more advanced cognitive and language abilities. In contrast, caregiver stress attenuated this relationship, such that higher quality caregiver strategies were less predictive of joint engagement at higher levels of stress.

**Discussion:**

These findings suggest that the impact of caregiver strategies on engagement is shaped by both toddler developmental capacities and caregiver well-being, highlighting the importance of tailoring caregiver-mediated interventions to account for individual differences in the toddler - caregiver dyad to optimize joint engagement outcomes.

## 1 Introduction

Play-based interactions between caregivers and toddlers provide a primary context through which children acquire social, communicative, and cognitive skills. During dyadic interactions, moments of joint engagement—when both the caregiver and child are actively involved with the same object or activity—create rich opportunities for learning and social communication (1, 2). Joint engagement is characterized by sustained, shared focus between social partners and the coordination of attention and communicative behaviors around a common referent (3). Despite its importance, the degree to which caregivers and toddlers achieve joint engagement varies considerably, and factors contributing to this variability remain largely understudied.

### 1.1 Joint engagement in early development

Toddlers with autism spectrum disorder (ASD) often experience social communication challenges, including delays in the development of joint attention gestures and reduced time spent in joint engagement relative to typically developing peers (2, 4, 5). Joint attention refers to specific social communicative behaviors (e.g., pointing, showing, or gaze coordination) used to direct and share attention with a social partner toward an object or event (6). In contrast, joint engagement refers to a broader interactional state in which two individuals share involvement with the same object, activity, or event (4).

Joint engagement is particularly important in early autism because it provides the social context in which young children can practice reciprocal social interactions, coordinate attention with others, and participate in meaningful communicative exchanges that support language learning. During periods of joint engagement, caregivers can follow the child's focus of attention, provide language input, scaffold play, and create opportunities for communication. Consistent with this framework, prior research suggests that joint engagement may serve as an important mechanism supporting social communication development in this population, with longer periods of shared engagement associated with later expressive and receptive language and broader social communication outcomes among autistic children (2, 7–9).

### 1.2 Sources of variability in joint engagement

At the child level, age, early cognitive and language skills may influence a toddler's capacity to initiate and sustain joint engagement with a caregiver. Research in typically developing children suggests that developmental gains in social attention, communication, and play skills may support longer periods of joint engagement with caregivers and others (3, 10). In terms of cognitive abilities, skills related to attention information processing and symbolic understanding may support the coordination of attention during shared activities (11). Sustained attention may enable toddlers to remain engaged with an activity long enough to share their focus with caregivers. Information-processing abilities may support the interpretation of social and environmental cues during a shared interaction. Similarly, symbolic understanding may allow children to play in more flexible ways and recognize meanings with objects and actions. Although these processes have been studied broadly in typically developing children, related findings have been observed in autistic children.

Among autistic children, joint engagement has been associated with subsequent language and social communication development, suggesting that periods of shared engagement may provide an important context for communication growth (7, 8, 12). Research involving toddlers at elevated likelihood of autism also suggests that episodes of joint engagement provide important contexts for caregiver language input, including object labeling, which may support language learning opportunities during interactions (13).

Taken together, the literature suggests a potentially bidirectional relationship between cognitive and communication abilities and joint engagement. Stronger language skills may support children's ability to interpret caregiver cues and participate in reciprocal interactions, potentially facilitating initiations of joint engagement that last for longer periods of time. Toddlers with more limited language abilities may initiate joint engagement less often than same aged peers, or may engage in shorter episodes of joint engagement, contributing to variability in joint engagement across dyads. At the same time, sustained periods of joint engagement may, in turn, provide increased opportunities for social communication and language growth.

At the caregiver level, parenting stress may also contribute to variability in joint engagement between child-caregiver dyads. Elevated stress has been associated with differences in interaction quality and responsiveness during caregiver-child interactions (14). Given that joint engagement depends on the caregiver's ability to follow the child's lead, respond, and sustain coordinated attention, high levels of stress may reduce the frequency or duration of joint engagement episodes. Although caregiver stress is frequently examined as an outcome of intervention or a predictor of caregiver behavior more broadly (15, 16), its direct role in shaping joint engagement quality or duration during naturalistic interactions warrants further examination. Together, child developmental characteristics and caregiver well-being may independently and jointly shape the conditions under which joint engagement is achieved and sustained. Understanding these sources of variability is important for explaining differences in social communication trajectories among young toddlers with autism and for informing more individualized approaches to supporting families.

### 1.3 Caregiver-mediated interventions and strategy use

Given the central role of joint engagement in early social communication development, intervention approaches increasingly emphasize the active involvement of caregivers within children's everyday environments. Current recommended practices highlight caregivers as critical agents of change and advocate for intervention to occur within naturalistic contexts, such as the home and family routines (17–19). Caregiver mediated interventions, in which caregivers are often coached or taught strategies, are a primary approach for promoting developmental skills within naturalistic settings. Consistent with these recommendations, caregiver-mediated interventions for young children with autism have expanded substantially and commonly target caregiver strategy use (e.g., following child's attentional focus, expanding on child communication) as mechanisms for increasing joint engagement and social communication (9, 20–22, 40, 41).

Although caregiver-mediated interventions have been shown to improve joint engagement and social communication outcomes at the group level (9, 20–22), substantial variability in treatment response has been documented across children. Studies examining developmental trajectories and intervention response have identified child characteristics, including age, cognitive and language abilities, as predictors of differential treatment response. For example, higher baseline cognitive and language abilities have been associated with greater gains following intervention, suggesting that children with stronger developmental skills may be better positioned to benefit from some caregiver-mediated approaches (23–26, 42). Emerging evidence further suggests that caregiver characteristics and behaviors may also contribute to differential treatment response and outcomes. For example, caregiver responsiveness and strategy implementation have been linked to improved treatment outcomes, whereas elevated parenting stress may create challenges for treatment implementation or participation (21, 24, 27). These findings highlight that the degree to which caregiver strategy use is associated with joint engagement may not be uniform across dyads. Examining whether toddler characteristics moderate the association between caregiver strategy use and joint engagement can help explain the variability in outcomes and perhaps inform more individualized and responsive approaches to supporting families of young toddlers with autism.

### 1.4 The current study

The present study is a secondary data analysis aimed to examine whether toddler age, cognitive abilities, language abilities (expressive and receptive), and caregiver stress attenuated or amplified the association between caregiver strategy use and joint engagement during caregiver–child interactions among toddlers with autism. We hypothesized that older toddlers, and toddlers with stronger cognitive and language abilities would strengthen the association between caregiver strategy use and joint engagement, while elevated caregiver stress would weaken the association.

## 2 Methods

### 2.1 Design

Data for the current study were from the baseline (entry) assessments of a randomized controlled trial intervention for infants and toddlers showing early signs of autism spectrum disorder (P50HD055784). The study was approved by the institutional review board at the university. Families were recruited from community-based healthcare services, early intervention providers, internet flyers and self-referrals. All families engaged in informed consent processes and provided written consent to participate.

### 2.2 Participants

A total of 80 toddlers and their caregivers were included in the present study (*M* age = 26.26 (months), SD = 7.08, range = 12–36 months). Children were included if they (a) were between 12 and 36 months of age, (b) had elevated scores on the Autism Diagnostic Observation Schedule-2 (28–30) and were judged by expert clinicians to have exhibited clear behavioral features of autism spectrum disorder, (c) were free from co-occurring neurological or genetic conditions (e.g., tuberous sclerosis complex), major physical or sensory impairments (e.g., cerebral palsy, blindness), or uncontrolled seizure activity, (d) completed a video-recorded 10 min caregiver-child interaction and clinical assessments and questionnaires at entry into study. The primary caregiver was determined through participation in the caregiver-child interaction (CCX).Elevated ADOS scores were defined as overall total scores consistent with the “mild-to-moderate range of concern” cut-off or higher for those administered the Toddler Module, or overall total scores consistent with the “autism spectrum” cut-off or higher for those administered Module 1 or 2. ADOS-2 scores were used for inclusion purposes into this study and were not used in subsequent analyses.

As this study is a secondary data analysis, *a priori* power analyses were established for the original intervention study.

### 2.3 Measures

The present study uses parent questionnaires and direct child assessments. Assessments were administered and coded by a research team consisting of trained graduate students, postdoctoral students, and research staff. For coded measures, blinded research staff were required to reach 90% reliability, measured using interclass correlation coefficients (ICC) for each variable of interest prior to coding.

#### 2.3.1 Demographic questionnaire

Primary caregivers of participating toddlers (i.e., parents, legal guardians) completed a demographic form at entry into the study. This form included child, parent, and family-level information such as the parent's education and income levels.

#### 2.3.2 Autism diagnostic observation schedule-2nd edition

The ADOS-2 (28) is a semi-structured play-based assessment used to aid in the diagnosis of autism spectrum disorder. Toddlers who were 30 months of age or younger at the time of the assessment were administered the ADOS Toddler Module (29), whereas those aged 31 months or older were administered Module 1 or 2, depending on their language level. Using diagnostic algorithms determined by the child's chronological age and language level, ADOS total scores may be converted into calibrated severity scores (CSS) ranging from “1” to “10”, where higher scores reflect higher levels of autism features. Scores from the ADOS-2 were used solely for inclusion purposes (30).

#### 2.3.3 Mullen scales of early learning (MSEL)

The Mullen Scales of Early Learning (MSEL) is a standardized developmental assessment of cognitive and motor functioning for young children up to 68 months of age and is frequently used to assess cognitive abilities of infants and children with neurodevelopmental conditions or delays. The Mullen includes five scales assessing gross motor skills, fine motor skills, visual reception, receptive language, and expressive language. Analyses included the child's age equivalent scores for the language subscales (expressive language and receptive language scores), as well as the combined Early Learning Composite score, which is the general cognitive ability score comprised of each of the subscales (31).

#### 2.3.4 Caregiver-child play interaction (CCX)

Caregiver-child dyads completed a 10-min free-play interaction at entry (20). Caregivers were asked to engage with their child as they normally would at home using a standard set of toys, spanning across developmental play levels (9).

The CCX was coded via ratings for the quality and appropriateness of caregiver strategy implementation during the free-play interactions and was quantified as in Gulsrud et al. (21). Caregiver strategies (30 items) were grouped into six domains aligned with the JASPER intervention: (a) supports for engagement and regulation (engagement and regulation) and (b) environmental arrangement, (c) balancing imitating and modeling of play acts (imitation and modeling) and (d) establishing play routines (play routines), (e) responding to and expanding nonverbal communication (nonverbal communication) and (f) responding to and expanding verbal communication (language). Strategies were rated on a scale of 0–5, with 0 reflecting no or poor implementation of strategies/behaviors and 5 reflecting consistent, fluent, and appropriate use of all strategies/behaviors within the domain. A total percentage score for caregiver strategy implementation was used for analyses. Inter-rater reliability was assessed via intra-class correlations for each strategy domain and ranged between .91 and 1.00 (*M* = .93). Throughout coding, frequent reliability checks (∼25% of videos) occurred, wherein videos were double coded across coders to ensure reliability was maintained.

#### 2.3.5 Engagement states

Engagement states were coded during the CCX) using a state-based coding system. A state was recorded when a child remained in one of the several mutually exclusive categories for at least three consecutive seconds. Engagement states include (a) unengaged (e.g., lack of involvement with people or objects, for example wanders or looks around), (b) onlooking (child observes the play partner, but does not participate in play), (c) person engaged (e.g., attends to a person only, participating in a song or social game with no object), (d) object engaged (e.g., child focuses exclusively on an object without noticing person), (e) supported joint engaged (child demonstrates awareness of both the social partner and the shared activity) and (f) coordinated joint engaged (e.g., child drives the interaction by coordinating both the play partner and the shared activity). The total time jointly engaged is calculated as the combined duration of both the supported joint engaged and coordinated joint engaged states, and was the primary variable used for analyses [adapted from (32)].

#### 2.3.6 Parenting stress index-fourth edition (PSI-4)

Caregiver stress was assessed using the Parenting Stress Index–Fourth Edition (PSI–4) (33), a widely used caregiver-report measure designed to evaluate stress associated with the parenting role. The PSI-4 assesses multiple domains of parenting stress related to both child and caregiver characteristics. The child domains include (a) adaptability, (b) acceptability, (c) demandingness, (d) distractibility/hyperactivity, (e)mood and (f) reinforces parent. Parent domains include: (a) attachment, (b) competence (c) depression, (d), (e)isolation, (f) role restriction, and (g) spouse/partner relationship. Caregivers rated items on a Likert-type scale, with higher scores indicating greater parenting stress. The PSI-4 has demonstrated strong psychometric properties, including good internal consistency and validity, and has been used extensively in studies of families of young children, including those with developmental disabilities (34). The three primary variables used for analysis include the total stress composite score, the parent domain subscale score, and the child domain subscale score.

### 2.4 Quantitative analyses

All statistical analyses were conducted in R (R Core Team, 2023.12.0+369). Continuous variables were evaluated for statistical assumptions, including normality (*Q*–*Q* plots), linearity (inspection of residual plots), and the presence of outliers (Cook's distance). To examine study hypotheses, exploratory *post hoc* analyses consisting of a series of multiple linear regression models were conducted to predict joint engagement from caregiver strategy use. Because these analyses were conducted using baseline data from a randomized intervention trial, they should be considered exploratory. The original trial was powered to detect group differences across intervention conditions, with a total sample size of 80 participants, and was not specifically powered for the present moderation analyses. In each model, caregiver strategy use, the moderator variable, and the interaction term were entered simultaneously. Potential moderators were tested in separate models and included (1) child age, (2) cognitive functioning (Early Learning Composite), (3) expressive language, (4) receptive language, (5) total parenting stress, (6) parent-domain stress, and (7) child-domain stress. Child age was included as a covariate in all models except when age was examined as the moderator. To correct for multiple testing, an adjusted cut off (*α* = 0.025) will be utilized to account for the two outcome variables (caregiver strategies and joint engagement).

## 3 Results

All model assumptions were met. Residuals were approximately normally distributed, visual inspections of diagnostic plots indicated no meaningful violations of linearity or homoscedasticity, and no influential outliers were detected based on Cook's distance.

### 3.1 Participant descriptives

Participant characteristics are displayed in Table 1. The final sample included 80 toddlers (75.3% male; *M* age = 26.26 (months), SD = 7.08, range = 12–36 months).

**Table 1:** Demographics.

Variable	Value	Sample
		Mean (SD) or *N* (%)
		*N* = 80
Child demographics		
Child age	(months)	26.26 (7.08)
Child's sex	Male	61 (75.3%)
	Female	19 (23.5%)
Child's race	Asian	18 (22.2%)
	Black	2 (2.5%)
	White	39 (48.1%)
	Other	20 (24.7%)
	Do not wish to disclose	1 (1.3%)
Child's ethnicity	Hispanic/Latino	15 (18.5%)
	Not Hispanic/Latino	63 (77.8%)
	Do not wish to disclose	2 (2.5%)
Mother demographics		
Mother's age	(years)	37.25 (4.80)
Mother's race	Asian	24 (29.6%)
	Black	3 (3.7%)
	White	39 (48.1%)
	Other	13 (16%)
	Do not wish to disclose	1 (1.3%)
Mother's ethnicity	Hispanic/Latino	12 (14.8%)
	Not Hispanic/Latino	66 (81.5%)
	Do not wish to disclose	2 (2.5%)
Father demographics		
Father's age	(years)	40.58 (6.38)
Father's race	Asian	19 (23.5%)
	Black	2 (2.5%)
	White	50 (61.7%)
	Other	8 (9.9%)
	Do not wish to disclose	1 (1.3%)
Father's ethnicity	Hispanic/Latino	9 (11.1%)
	Not Hispanic/Latino	69 (85.2%)
	Do not wish to disclose	2 (2.5%)
Primary caregiver demographics		
Primary caregiver type	Mother only	48 (60.0%)
	Father only	2 (2.5%)
	Both parents	22 (27.5%)
	Mother and Nanny	2 (2.5%)
	Mother, father, and Nanny	4 (5.0%)
	Nanny only	1 (1.3%)
	Do not wish to disclose	1 (1.3%)
Baseline scores		
Mullen (age equivalent in months)	Visual reception	20.93 (9.02)
	Fine motor	21.15 (6.18)
	Receptive language	17.20 (9.86)
	Expressive language	17.38 (9.73)
	ELC	72.80 (24.27)
Caregiver strategies		28% (11%)

### 3.2 Age as a moderator

**Figure 1:**
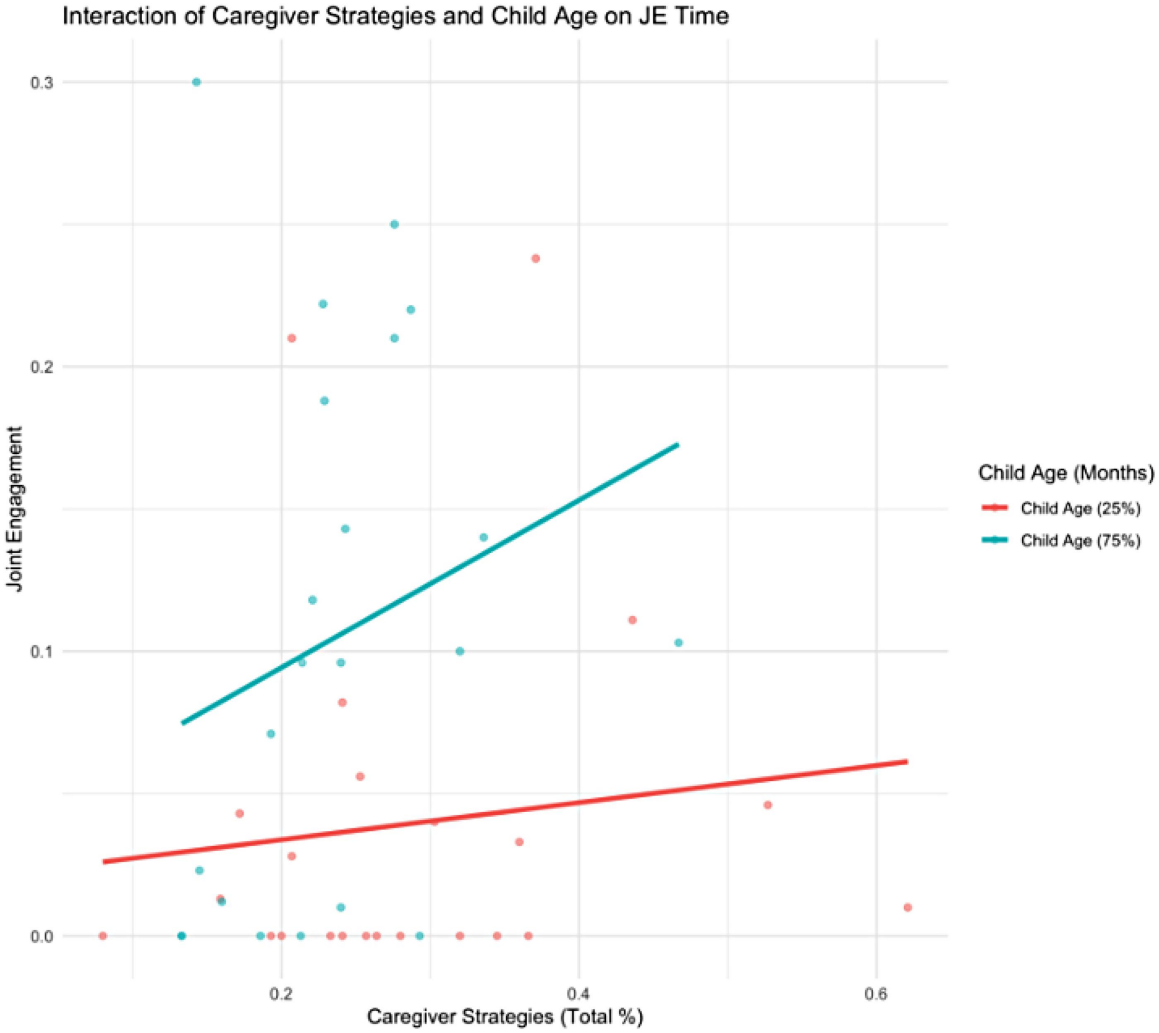
Child Age moderates the association between caregiver strategies and joint engagement (model 1).

A multiple linear regression model examined whether child age moderated the association between caregiver strategies and joint engagement. The overall model was significant (*Adjusted R*^2^ = .246, *p* < .001; Table 2). The interaction between caregiver strategies and age was significant **(***β* = 1.101, *p* = .015), indicating that the effect of caregiver strategies on engagement is strengthened when child age increases (Figure 1).

**Table 2:** Regression matrix (model 1: predicting engagement with caregiver strategies, moderated by child age).

Model	Predictor	Unstandardized Coefficients		Standardized coefficients	*t* value	Sig.
		B	Std. Error	Beta (*β*)		
1	(Constant)	0.114	0.130		0.882	.381
	Caregiver strategies	−0.666	0.455	−0.574	−1.462	.148
	Age	−0.005	0.005	−0.297	−1.127	.263
	Caregiver strategies × age	0.043	0.017	1.101	2.499	.015*

Model summary.

*R*^2^ = .274.

Adjusted *R*^2^ = .246.

*F*(3, 76) = 9.57, *p* < .001.

*p < .05.

### 3.3 Cognitive ability as a moderator

**Figure 2:**
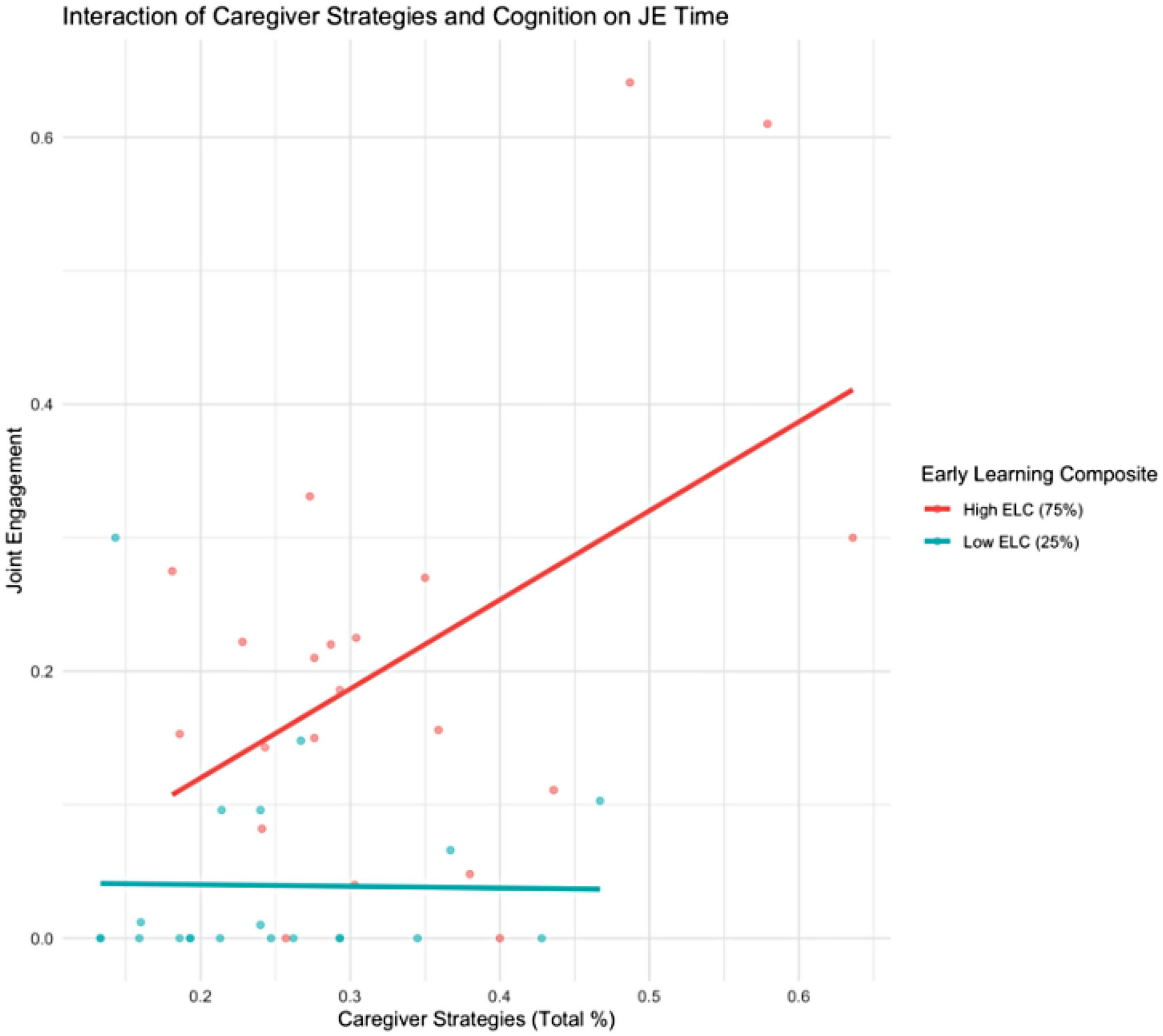
Early learning composite moderates the association between caregiver strategies and joint engagement (model 2).

The model examining cognitive ability as a moderator was significant (*Adjusted R*^2^ = .469, *p* < .001; Table 3). Child age was a significant predictor in this model **(***β* = 0.274, *p* = .001). The interaction between caregiver strategies and cognitive functioning was significant **(***β* = 1.108, *p* = .008), indicating that the effect of caregiver strategies on engagement increased among children with higher cognitive abilities, despite child age (Figure 2). To address potential overlap between language measures and the ELC, analyses were repeated using a nonverbal developmental composite derived from the Visual Reception and Fine Motor domains. The pattern of findings remained unchanged.

**Table 3:** Regression matrix (model 2: predicting engagement with caregiver strategies, moderated child early learning composite with child age as a covariate).

Model	Predictor	Unstandardized coefficients		Standardized coefficients	*t* value	Sig.
		*B*	Std. Error	Beta (*β*)		
7	(Constant)	−0.050	0.106		−0.475	.636
	Caregiver strategies	−0.587	0.321	−0.506	−1.831	.071
	Early learning composite	−0.001	0.001	−0.112	−0.465	.643
	Age	0.005	0.001	0.274	3.311	.001*
	Caregiver strategies × early learning composite	0.011	0.004	1.108	2.746	.008*

Model summary.

*R*^2^ = .496.

Adjusted *R*^2^ = .469.

*F*(4, 75) = 18.47, *p* < .001.

*p < .05.

### 3.4 Language abilities as a moderator

**Figure 3:**
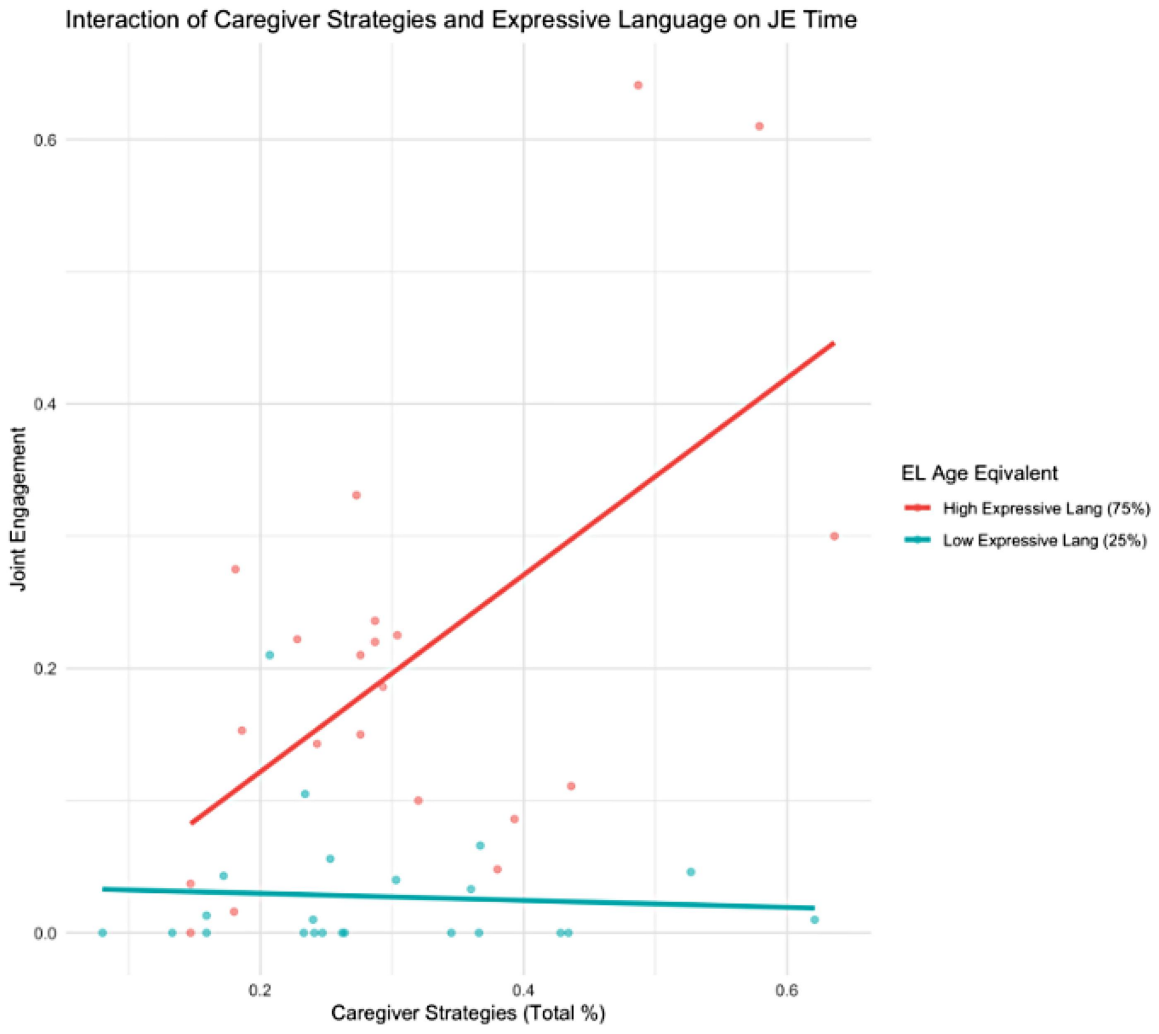
Expressive language moderates the association between caregiver strategies and joint engagement (model 3).

Both expressive and receptive language abilities were analyzed as potential moderators in the relationship between caregiver strategies and joint engagement. The model examining expressive language as a moderator was significant (*Adjusted R*^2^ = .475, *p* < .001; Table 4). The interaction between caregiver strategies and expressive language was significant **(***β* = 1.099, *p* < .001), indicating that the effect of caregiver strategies on engagement was stronger among children with higher expressive language abilities, despite child age (Figure 3).

**Table 4:** Regression matrix (model 3: predicting engagement with caregiver strategies, moderated child expressive language with child age as a covariate).

Model		Unstandardized coefficients		Standardized coefficients	*t* value	Sig.
		*B*	Std. error	Beta (*β*)		
5	(Constant)	0.036	0.071		0.502	.617
	Caregiver strategies	−0.310	0.196	−0.267	−1.584	.117
	Expressive language	−0.004	0.003	−0.339	−1.477	.144
	Age	0.002	0.002	0.084	0.917	.362
	Caregiver strategies × expressive language	0.038	0.010	1.099	3.896	<.001*

Model summary.

*R*^2^ = .502.

Adjusted *R*^2^ = .475.

*F*(4, 75) = 18.87, *p* < .001.

*p < .05.

**Figure 4:**
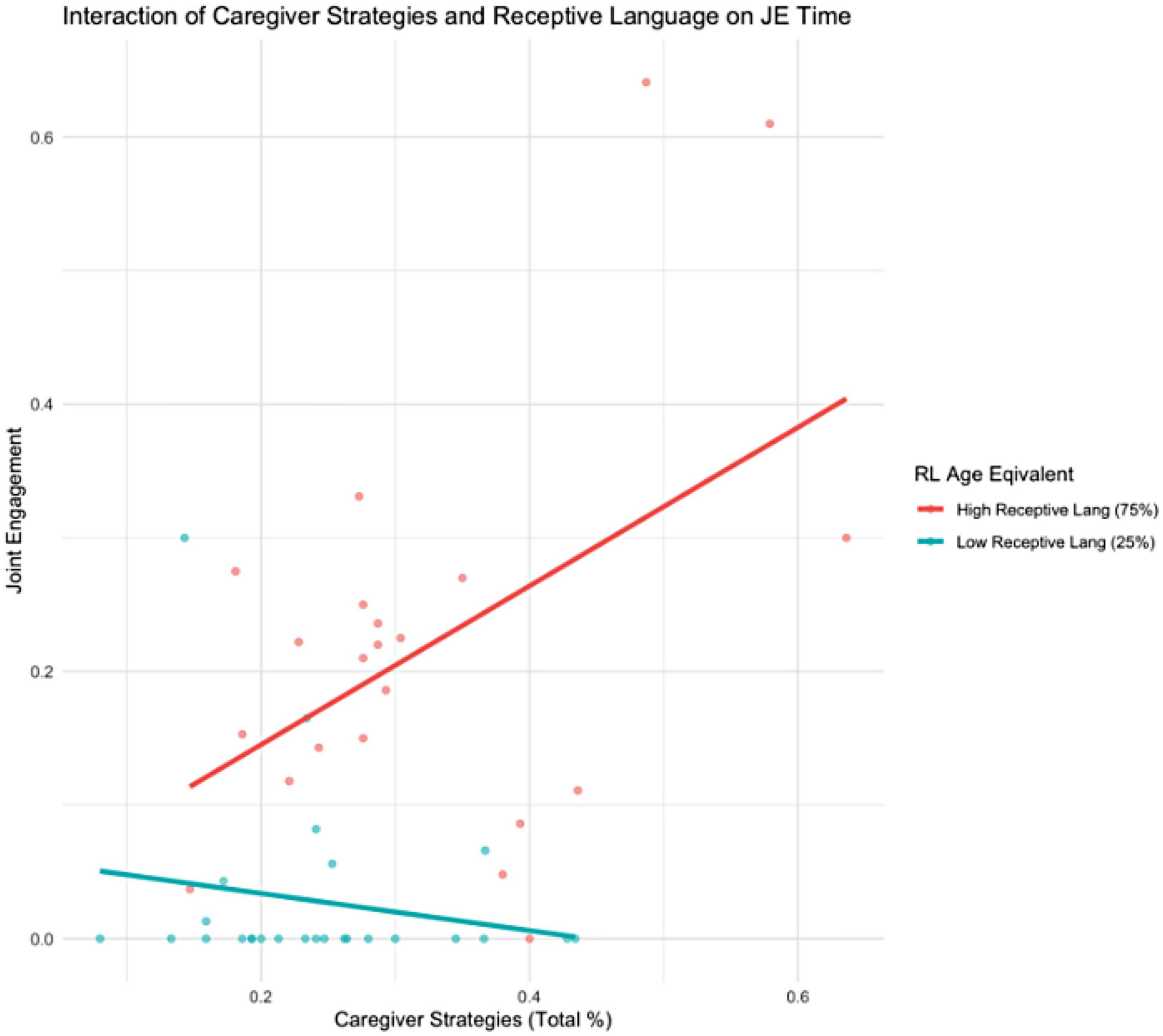
Receptive language moderates the association between caregiver strategies and joint engagement (model 4).

The receptive language model was also significant (*Adjusted R*^2^ = .462, *p* < .001; Table 5). A significant interaction appeared between caregiver strategies and receptive language **(***β* = 1.066, *p* = .001), suggesting that the effect of caregiver strategies on engagement was strengthened for children with higher receptive language skills, despite child age (Figure 4).

**Table 5:** Regression matrix (model 4: predicting engagement with caregiver strategies, moderated child receptive language with child age as a covariate).

Model		Unstandardized coefficients		Standardized coefficients	*t* value	Sig.
		*B*	Std. Error	Beta (*β*)		
6	(Constant)	0.044	0.073		0.612	.524
	Parent strategies	−0.385	0.212	−0.332	−1.814	.074
	Receptive language	−0.003	0.003	−0.227	−0.961	.340
	Age	0.002	0.002	0.100	1.084	.282
	Parent strategies × receptive language	0.033	0.010	1.066	3.418	.001*

Model summary.

*R*^2^ = .489.

Adjusted *R*^2^ = .462.

*F*(4, 75) = 17.95, *p* < .001.

*p < .05.

### 3.5 Parenting stress as a moderator

**Figure 5:**
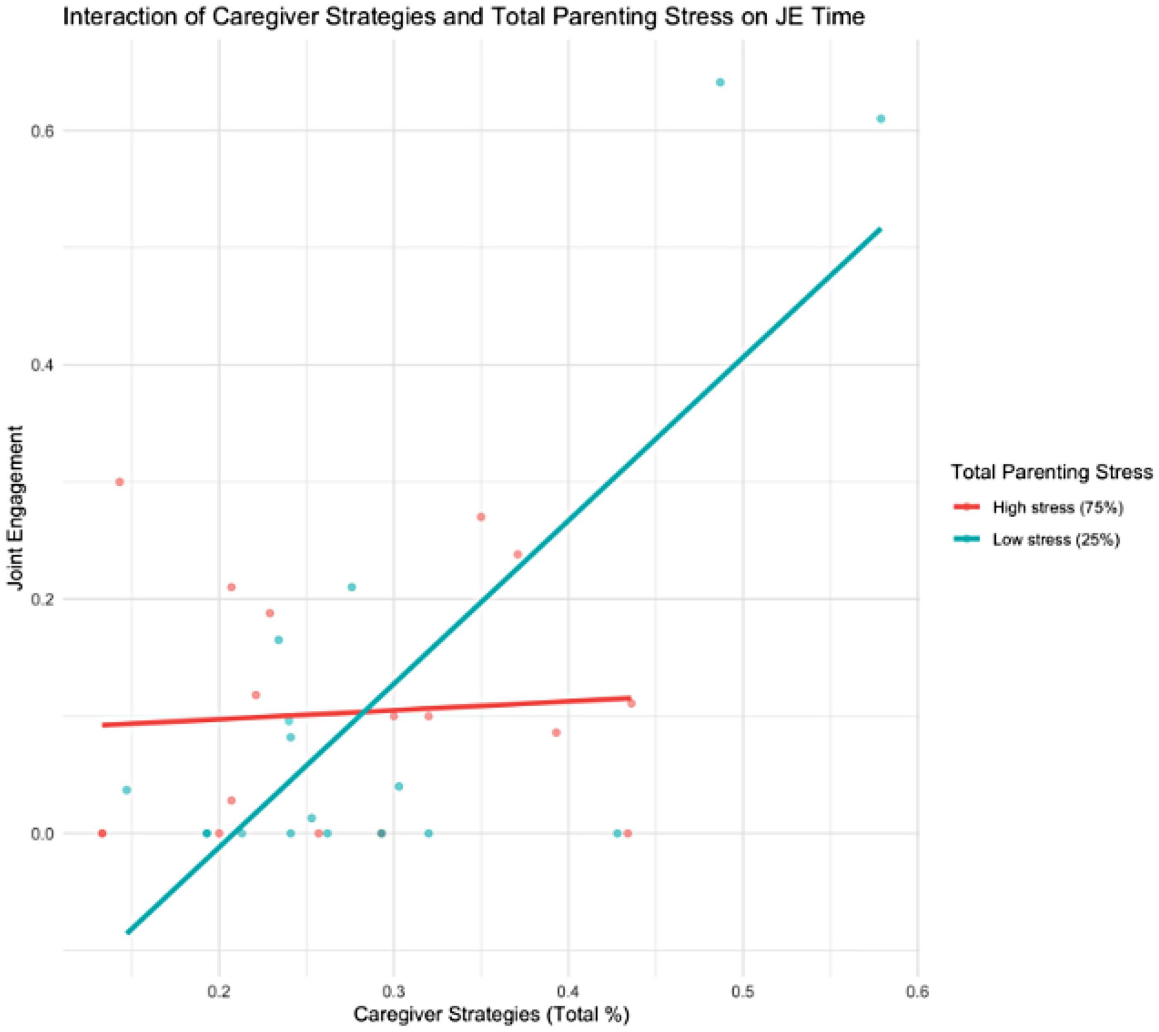
Total caregiver stress moderates the association between caregiver strategies and joint engagement (model 5).

**Figure 6:**
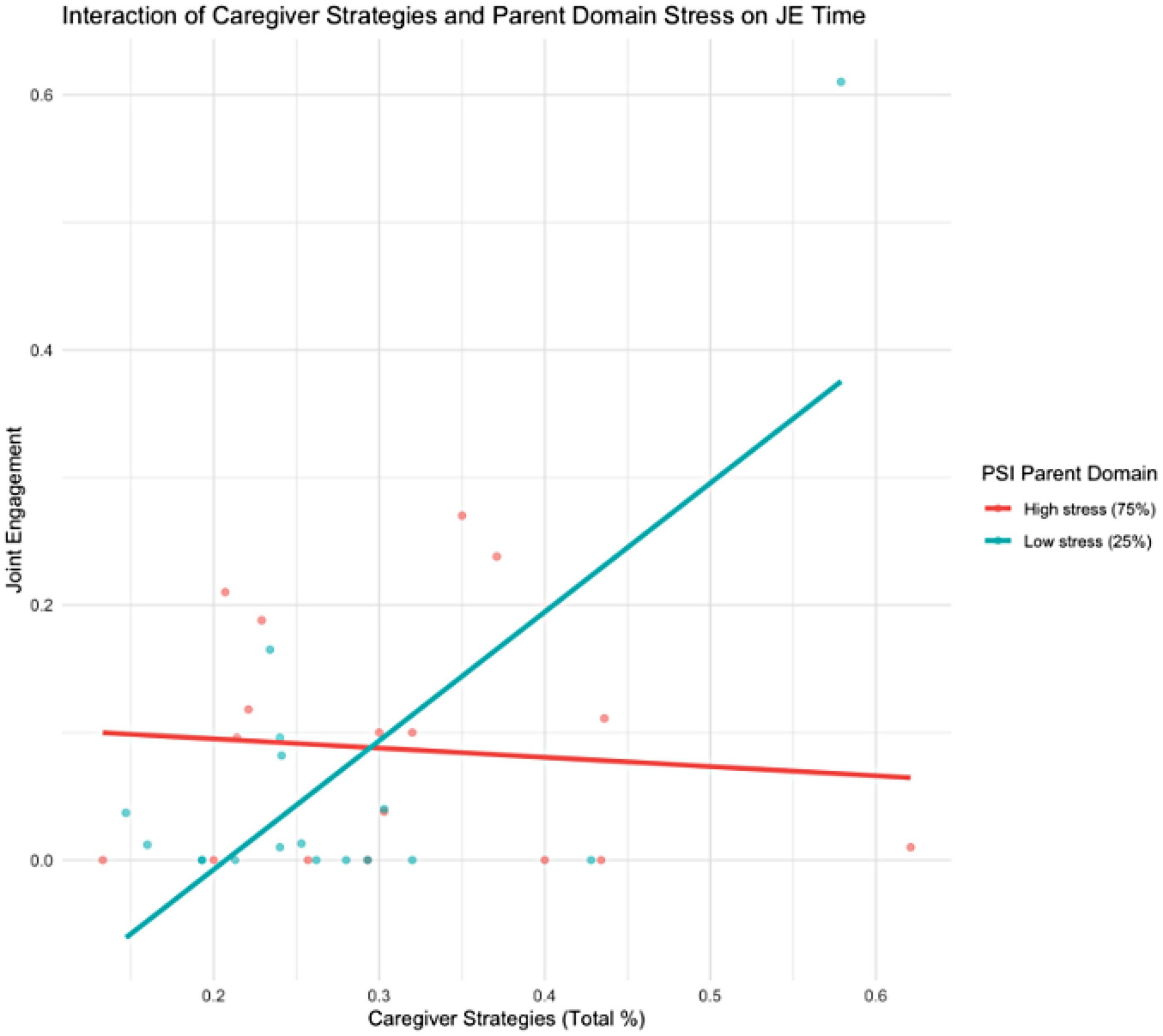
Parent domain stress moderates the association between caregiver strategies and joint engagement (model 6).

**Figure 7:**
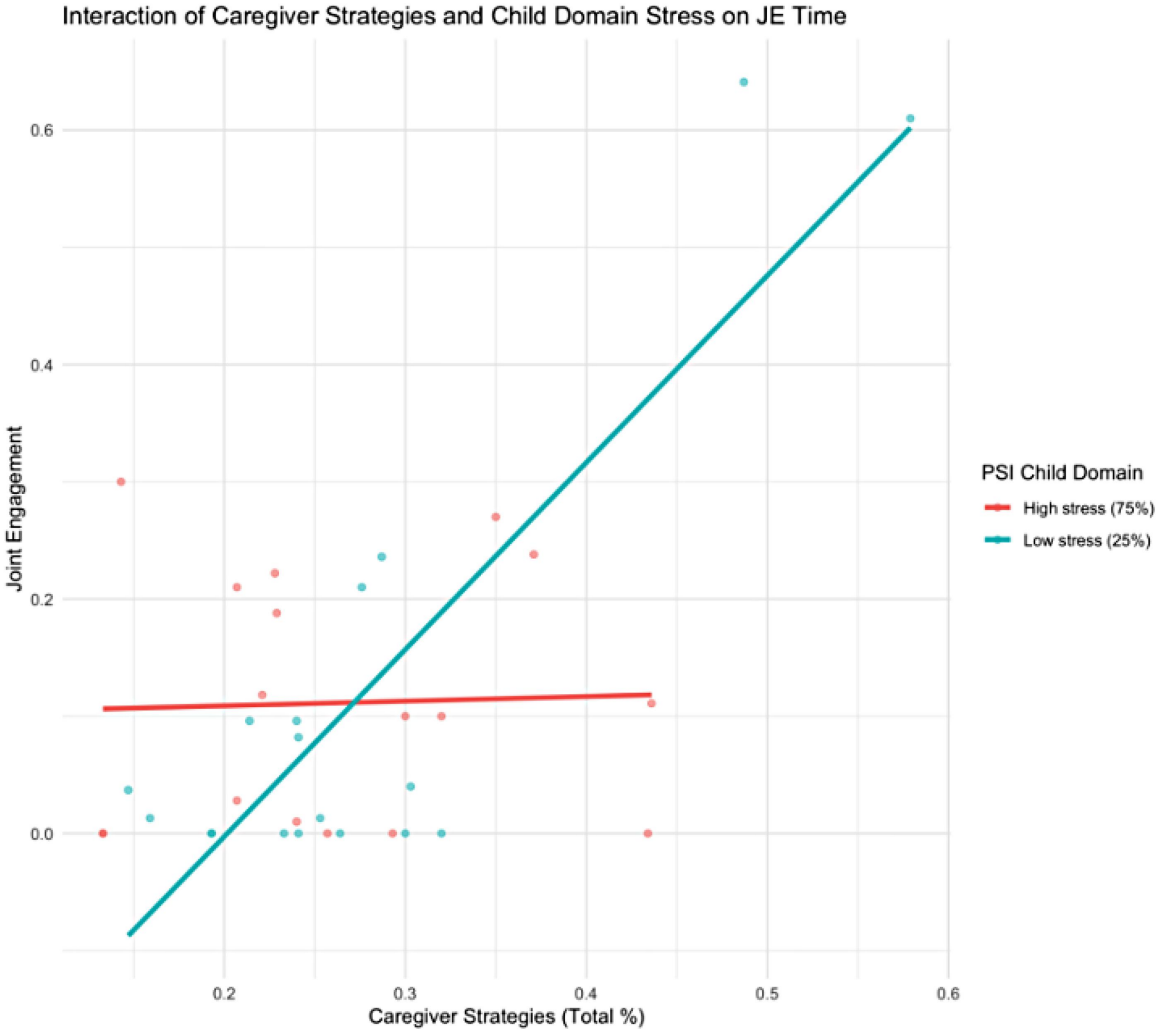
Child domain stress moderates the association between caregiver strategies and joint engagement (model 7).

Models examining total parenting stress, parent domain stress and child domain stress were significant (Total Parenting Stress: *Adjusted R*^2^ = .328, *p* < .001; Table 6; Parent Domain Stress: *Adjusted R*^2^ = .286, *p* < .001; Table 7; Child Domain Stress: *Adjusted R*^2^ = .312, *p* < .001; Table 8). Total parenting stress **(***β* = 1.044, *p* = .002) and caregiver strategies **(***β* = 2.547, *p* < .001)-were significant predictors in the total parenting stress model (Table 2). The interaction between caregiver strategies and total parenting stress was significant, despite child age **(***β* = −2.440, *p* < .001), indicating that the effect of caregiver strategies on engagement is weakened when total parenting stress increases (Figure 5). Results for domain-specific stress were consistent with the total stress model. Both parent and child domain stress significantly predicted joint engagement and moderated the association between caregiver strategies and engagement, such that the positive effect of caregiver strategies was attenuated at higher levels of stress (Figures 6, 7).

**Table 6:** Regression matrix (model 5: predicting engagement with caregiver strategies, moderated by total parenting stress with child age as a covariate).

Model		Unstandardized coefficients		Standardized coefficients	*t* value	Sig.
		*B*	Std. error	Beta (*β*)		
2	(Constant)	−0.858	0.215		3.985	<.001*
	Caregiver strategies	3.130	0.748	2.547	4.186	<.001*
	Parenting stress (total)	0.003	0.001	1.044	3.233	.002*
	Child age	0.005	0.002	0.236	2.271	.027
	Caregiver strategies × parenting stress	−0.010	0.003	−2.440	3.565	<.001*

Model summary.

*R*^2^ = .368.

Adjusted *R*^2^ = .328.

*F*(4, 63) = 9.186, *p* < .001.

*p < .05.

**Table 7:** Regression matrix (model 6: predicting engagement with caregiver strategies, moderated parent domain stress with child age as a covariate).

Model		Unstandardized coefficients		Standardized coefficients	*t* value	Sig.
		*B*	Std. error	Beta (*β*)		
3	(Constant)	−0.670	0.195		3.434	.001*
	Caregiver strategies	2.433	0.681	1.980	3.575	<.001*
	Parent domain stress	0.004	0.001	0.796	2.606	.011*
	Child Age	0.005	0.002	0.275	2.647	.010*
	Caregiver strategies × parent domain stress	−0.014	0.005	−1.871	2.886	.005*

Model summary.

*R*^2^ = .329.

Adjusted *R*^2^ = .286.

*F*(4, 63) = 7.709, *p* < .001.

*p < .05.

**Table 8:** Regression matrix (model 7: predicting engagement with caregiver strategies, moderated child domain stress with child age as a covariate).

Model		Unstandardized coefficients		Standardized coefficients	*t* value	Sig.
		*B*	Std. Error	Beta (*β*)		
4	(Constant)	−0.734	0.193		−3.800	<.001*
	Caregiver Strategies	2.671	0.671	2.174	3.981	<.001*
	Child domain stress	0.005	0.002	1.025	2.981	.004*
	Child age	0.004	0.002	0.217	1.977	.052
	Caregiver strategies × child domain stress	−0.018	0.005	−2.022	−3.300	.002*

Model summary.

*R*^2^ = .353.

Adjusted *R*^2^ = .312.

*F*(4, 63) = 8.602, *p* < .001.

*p < .05.

## 4 Discussion

The present findings indicate that both child developmental characteristics (age, cognition, and language) and caregiver stress were associated with differences in the strength of the relation between caregiver strategy use and joint engagement during caregiver-child interactions among toddlers with autism. Specifically, the relationship between caregiver strategy use and joint engagement was strongest for children who were older and who demonstrated stronger cognitive and language abilities. A similar pattern was observed under conditions of lower caregiver stress. Together, these findings highlight the importance of considering both child and caregiver characteristics when evaluating and implementing caregiver-mediated interventions.

This study found that toddler's cognitive abilities moderated the relation between caregiver strategy use and joint engagement, with stronger associations observed for children with higher cognitive scores. Children with stronger cognitive abilities may be better able to manage the attentional demands of shared activities, potentially making it more likely that caregiver strategies (such as following the child's lead or expanding play) are associated with longer episodes of joint engagement. This interpretation is consistent with prior work linking cognitive development to the emergence of coordinated joint attention and symbolic play (3, 6), processes that are closely tied to social communication development in children with autism (6, 35).

Similarly, expressive and receptive language abilities significantly moderated the association between caregiver strategy use and joint engagement, such that caregiver strategies were strongly associated with engagement among toddlers with higher baseline language skills. Children with stronger expressive skills may respond more frequently to caregiver bids and initiate communication that helps sustain the interaction (e.g., commenting and sharing during play), while receptive language abilities may facilitate understanding of caregiver cues and shared attention bids. As toddlers' communication abilities develop, they become better able to participate in reciprocal exchanges with their caregivers, further supporting joint engagement during interactions.

Importantly, these findings align with transactional models of early social development, suggesting that joint engagement emerges from continuous exchanges between child and caregiver. While caregiver strategies are often treated as universally effective, the present results suggest that the association between caregiver strategy use and joint engagement may be stronger among children with higher language and cognitive abilities, and weaker among children with more limited developmental skills.

In addition to toddler characteristics, caregiver stress emerged as a significant moderator of the association between caregiver strategy use and joint engagement. Across models examining both total caregiver stress and child-related stress, caregiver strategies were more strongly associated with higher levels of joint engagement under conditions of lower stress, with this association weakening as stress increased.

Elevated stress may influence caregiver-child interactions in ways that extend beyond the use of specific intervention strategies. Although caregivers experiencing higher levels of stress may still demonstrate appropriate strategy use during play interactions, stress may affect broader aspects of the caregiver-child relationship, including emotional availability, synchrony, and overall quality of shared experiences (36, 37). These broader relational processes may help explain why caregiver strategies were less strongly associated with joint engagement under conditions of elevated stress.

Stress related specifically to child characteristics may further shape how caregivers interpret and respond to their toddler's behavior, potentially influencing moment-to-moment strategy use and overall interaction quality. Although caregiver stress is frequently examined as an outcome of intervention, emerging work in caregiver-mediated interventions suggests that parenting stress may also influence how caregivers engage with and benefit from intervention strategies [e.g., (38)]. Notably, Schlink and colleagues examined slightly older toddlers (*M* = 30 months) whereas toddlers in the present study were younger (*M* = 26 months), suggesting that the influence of caregiver stress may emerge even earlier in development. The present findings therefore extend the literature by highlighting caregiver stress as a contextual factor associated with variability in the relation between caregiver strategy use and caregiver-child joint engagement. Collectively, these findings suggest that the effectiveness of caregiver strategies may depend on the intersection of child developmental readiness (e.g., cognitive and language abilities) and caregiver capacity (e.g., stress), factors that jointly shape toddlers' opportunities to participate in sustained joint engagement, a key context for early learning.

These findings have important implications for caregiver-mediated intervention models. Results suggest that the relation between caregiver strategy use and joint engagement may not be equally strong across all children and family contexts, particularly for children with lower language or cognitive abilities and for caregivers experiencing higher levels of stress. For these families, the findings raise the possibility that toddlers and caregivers may differ in the extent to which caregiver strategy use is related to joint engagement. This potentially highlights the need for additional research on individualized or intensive intervention approaches, strategies or techniques. Additionally, baseline caregiver and child characteristics may be associated with variability in intervention responsiveness, as where a caregiver or child begins (prior to intervention) matters to subsequent treatment gains [e.g., (39)].

Future research may examine whether incorporating additional supports for caregivers of children with lower developmental abilities, such as more explicit scaffolding, adapted strategies, or increased professional involvement. Similarly, future studies should evaluate whether addressing caregiver stress through supportive services, coaching, or stress-reduction components (such as mindfulness) strengthens the association between caregiver strategy use and joint engagement Emerging intervention approaches have begun to incorporate caregiver well-being and mental health supports into caregiver-mediated autism interventions. For example, programs integrating parent training with acceptance and commitment-based approaches have demonstrated improvements in caregiver implementation of intervention strategies (43). Other programs have incorporated supports targeting parental well-being, routines, and parenting practices alongside child intervention goals (21, 44, 45). Future research is needed to determine whether intervention approaches that account for child developmental abilities and caregiver well-being produce improved engagement outcomes.

Several limitations should be considered when interpreting these findings. First, child abilities and caregiver stress were measured at baseline, prior to intervention, limiting conclusions about how changes in these factors over time may be associated with caregiver strategy use and joint engagement. The use of observational measures (e.g., the CCX) during a single brief play interaction may not fully capture variability in caregiver behavior or caregiver-child joint engagement across everyday settings or routines. Caregiver stress was measured using caregiver self-report which may not fully reflect stress-related behaviors or other aspects of caregiver well-being. Future research would benefit from incorporating multiple methods and informants to more comprehensively assess caregiver stress. Additionally, there may be other factors important to the caregiver-child dyad that were not measured but that may influence interactions (e.g., parental beliefs, family resources, or other family stressors, etc.). Furthermore, participants were recruited as part of an intervention study and may not represent the broader population of families of toddlers with autism, potentially limiting generalizability of findings. Finally, the current sample consisted of young preverbal toddlers with autism within a relatively restricted age range, which may limit the generalizability to older, verbal, children. However, this focus on very young, preverbal toddlers is also a strength to the study, as few studies include children in this early stage of development with limited language skills. Given the increasing emphasis on early identification and intervention, understanding factors associated with caregiver-child interactions during this developmental period may provide important insight into mechanisms that support early social communication development. Finally, future research should examine longitudinal relations among caregiver strategies, child engagement, and changes in child and caregiver characteristics, as well as test whether tailoring intervention strategies based on baseline abilities and caregiver stress improves outcomes.

Collectively, these findings highlight the transactional, bidirectional nature of early caregiver–child interactions, demonstrating that child abilities and caregiver stress each moderate the observed association between caregiver strategies and joint engagement. Since joint engagement is a critical context for early learning and social communication development, factors associated with variability in the relation between caregiver strategy use and joint engagement may also be relevant to toddler's opportunities to benefit from everyday interactions. From an intervention standpoint, these results suggest that future research should examine whether tailoring intervention approaches based on child developmental abilities and caregiver stress improves engagement outcomes.

## Data Availability

The datasets presented in this study can be found in online repositories. The names of the repository/repositories and accession number(s) can be found below: NDAR. https://nda.nih.gov/.
